# “The Three Gorges” flap based on three tributaries—the bilateral deep inferior epigastric, superficial circumflex and external pudendal systems

**DOI:** 10.1093/jscr/rjaf408

**Published:** 2025-06-18

**Authors:** Nadia Hui Shan Sim, Allen Wei-Jiat Wong

**Affiliations:** Plastic, Reconstructive & Aesthetic Surgery Service, Sengkang General Hospital, 110 Sengkang E Way, Singapore 544886, Singapore; Plastic, Reconstructive & Aesthetic Surgery Service, Sengkang General Hospital, 110 Sengkang E Way, Singapore 544886, Singapore

**Keywords:** mega-flap, DIEP, SCIP, external pudendal artery

## Abstract

This study aims to identify a novel mega-flap from the abdominal region. The flap is an extension of the bipedicled DIEP to incorporate the bilateral superficial circumflex iliac perforator (SCIP) and external pudendal arterial systems. These arise from the common femoral artery, hence termed “The Three Gorges” flap. We utilized a novel indocyanine green fluorescence angiography method to evaluate the perfusion of this flap solely via the bilateral DIEP pedicles. Anatomical dissection of three cadavers was performed to map the perfusion zone of the flap via bilateral DIEP pedicles. Indocyanine green fluorescence angiography was employed to capture the perfusion in real time. The SCIP and superficial external pudendal systems can be perfused by the DIEP pedicle. A novel mega-flap based on three tributaries from bilateral DIEP, SCIP, and superficial external pudendal arterial systems can be supplied by bilateral DIEP pedicles.

## Introduction

The reconstruction of large and wide defects after oncological resection or post-traumatic injury requiring coverage remains a challenge. Options for reconstruction of massive defects are typically limited to the combined anterolateral and anteromedial (ALT-AMT) thigh flap [1, 2], latissimus dorsi (LD) flap and the bipedicled deep inferior epigastric perforator (DIEP) flap [3].

Adjacent to the DIEP territory are the superficial circumflex iliac artery perforator flap (SCIP) and the superficial external pudendal flaps. They are supplied by the superficial circumflex iliac artery (SCIA) and superficial external pudendal artery that arise from the external iliac artery. Individually, they are reliable and well-established reconstructive options. We postulate that the integration of these regional vascular systems into a single flap can potentially extend the coverage of the DIEP flap.

We introduce a novel free flap that combines the bilateral DIEP, SCIP, and the superficial external pudendal arterial systems. Thus adding an important option into our armamentarium for the reconstruction of massive defects. As the three arterial systems of the flap collectively arise from the external iliac artery, we term it the “Three Gorges” flap.

## Case report

**Figure 1:**
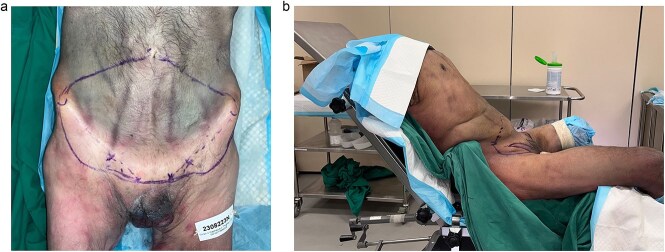
(a) The markings of the three gorges mega-flap are outlined. It incorporates the vascular territories of bilateral deep inferior epigastric artery (DIEA), superficial circumflex iliac artery (SCIA), and superficial external pudendal artery. (b) The specimen was positioned in jackknife to allow for a pinch test that determines the superior limit of the flap.

A commercially sourced adult male cadaver was used in the study. The inferior-lateral border of the conventional DIEP flap is marked beyond the inguinal ligament distal to bilateral groin creases and connected by a straight line that lies 2 cm below the pubic tubercle. The superior aspect of the flap is determined by a pinch test (Fig. 1a). This was aided by positioning the specimen in a jackknife position (Fig. 1b). A flap measuring 18 by 30 cm in size was marked.

**Figure 2:**
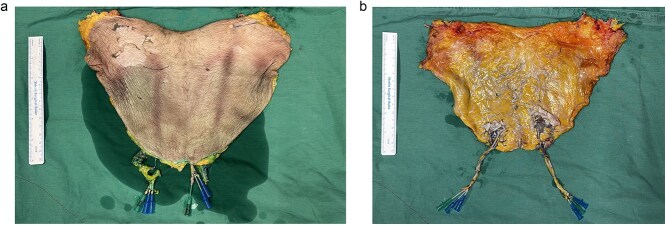
A 17 by 30 cm flap was raised. (a) Size of the skin paddle. (b) The underside of the flap.

The flap was raised in a lateral to medial fashion using bipolar cautery. The inferior incision was first made to raise bilateral SCIP territories. The superficial circumflex iliac artery and vein are traced to the origin of the external iliac and saphenous vein respectively and divided. The umbilicus was isolated and the superior incision was completed. Attention is focused on the DIEP territory. Dissection is continued until the lateral row perforators of the deep inferior epigastric system are encountered. The anterior rectus sheath was incised and deep inferior epigastric artery and vein were traced to the main pedicle and ligated. Both DIEAs were cannulated with an 18G cannula (Fig. 2a and b).

**Figure 3:**
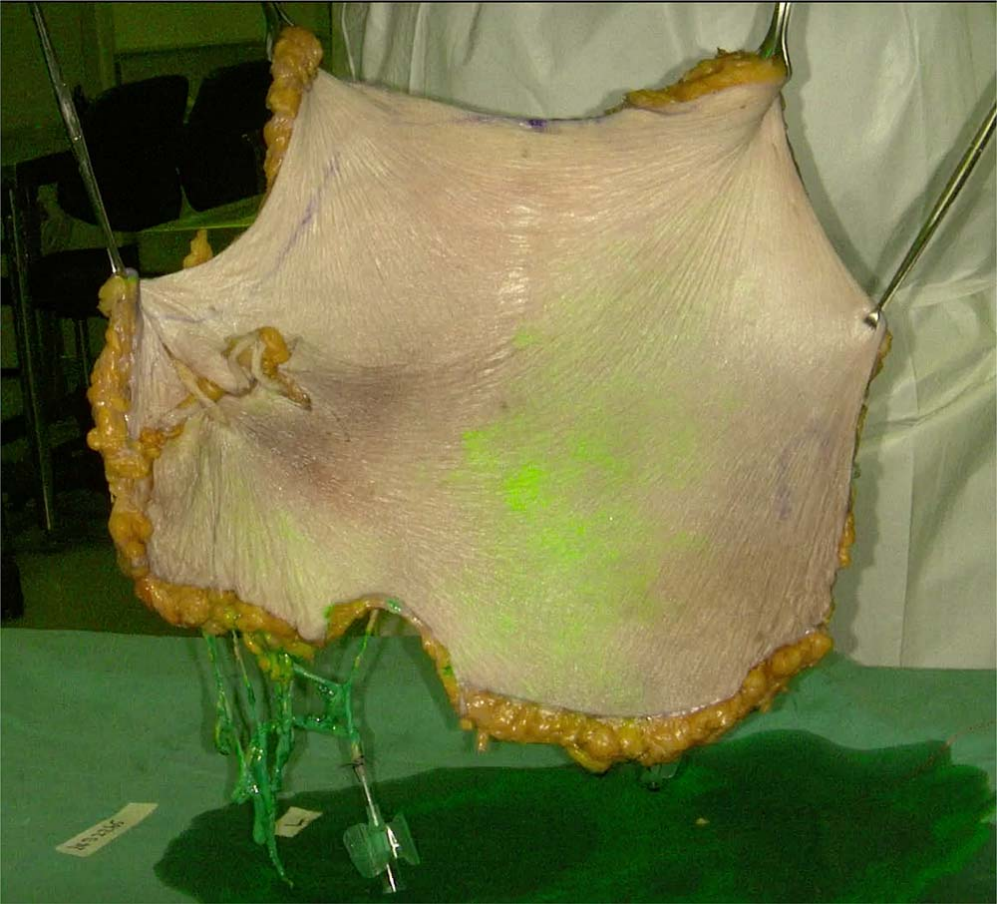
A novel vascular mapping technique is demonstrated using indocyanine green (ICG) angiography. The flap is suspended at its cranial end by tissue-grasping forceps. Both DIEA pedicles were injected in antigravitational manner with 10 ml of ICG solution and flushed with 20 ml of normal saline. Real-time imaging was captured with near-infrared camera.

The flap was suspended at its cranial end by tissue grasping forceps (Fig. 3). Both DIEAs were each injected concurrently with 10 ml of ICG solution (ICG Pulsion, Pulsion Medical System, Germany) (50 mg diluted in 10 ml of sterile water) and flushed with 20 ml of normal saline. This was done in an antigravitational manner to avoid potential ICG staining that may interfere with results. Real-time imaging was captured using fluorescence angiography with a near-infrared camera (Stryker SPY-PHI) (Supplementary Video 1).

**Figure 4:**
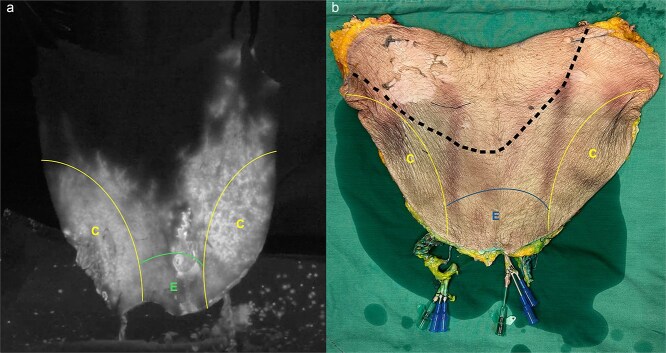
Side-by-side comparison of ICG fluorescent imaging of the flap perfusion against actual territory of the flap. (a) ICG imaging of the flap depicts complete perfusion of bilateral SCIP territory (C) and external pudendal artery territory (E). Fluorescence reached the most cranial aspect of the left DIEP flap territory. (b) Area of the right DIEP flap that was not fully perfused is marked out by dotted black lines.

Bilateral SCIP and superficial external pudendal territories were perfused. Fluorescence signal reached the most superior aspect of the left DIEP flap. The right DIEP territory was not fully perfused (Fig. 4).

## Discussion

The reconstruction of large and wide defects remains a challenge. The workhorse flaps (ALT-AMT flap [1, 4] and the LD flaps [5]) are disadvantaged by the limited dimensions of a single angiosome. The sacrifice of muscle compartments in free muscle transfer can result in additional morbidity.

Modern developments of combining flaps based on two or more separate but adjacent vascular territories have given us more options. Most recently, a combined SCIP with SIEA or DIEP flap has been described to overcome the size limitations of a single territory flap from the groin [6]. Such flaps can reach dimensions of 21 × 9 cm and 16 × 9 cm. However, the resultant flap has a skin paddle configuration that is “bipenate” with a short pedicle. Even modular mega-flaps that combines the SCIP, intercostal artery perforator to the thoracodorsal artery perforator flaps that allow reconstruction of defects up to 50 cm in length, involve difficult perforator to perforator anastomoses and prolonged staged reconstruction that are more suitable for long and narrow extremity defects [7]. Confronted with such limited reconstructive options, we sought to look for an alternative option with minimal donor site morbidity and reliable anatomy.

The use of the bipedicled DIEP flap has gained traction in recent years. Owing to its expansive surface area and minimal donor site morbidity, the application of the flap has extended beyond breast reconstruction and has been used to reconstruct massive defects in the extremities and head and neck. Successful large tissue reconstructions have been performed with bipedicled DIEP flaps with dimensions ranging from 20 × 8 cm to 50 × 17 cm [8]. Applying the perforasome concept, we hypothesize that perforators in the adjacent bilateral SCIP and superficial external pudendal artery territories can be captured through the DIEP interperforator flow mechanism [9]. By using ICG angiography, we demonstrated that majority of our megaflap (all six territories) may be perfused via bilateral DIEP pedicles. Supercharging or turbocharging the SCIP pedicle may enhance the perfusion of the flap.

Donor site morbidity is minimal as this is a perforator-based flap with no muscular sacrifice. To prevent wound dehiscence, the maximum height of the flap should be assessed with a pinch test and closure should be performed with repair of the fascial system with the patient in jackknife and hips flexed. A steep learning curve and prolonged operative duration may be a potential disadvantage to this flap.

We have developed a novel technique in perfusion studies for mapping vascular territory during cadaveric dissections. The use of colored dye in cadaveric vascular studies has been widely reported [10]. Compared to conventional dyes or methylene blue, ICG can outline a larger area when injecting equal volumes [11]. By conducting ICG fluorescent angiography after complete separation of the flap we were able to clearly visualize the perforasomes and the interperforator flow mechanism between territories (Supplementary Video 1). To avoid ICG leak that may cause unwanted staining and inaccurate results, we conducted dissection with meticulous “hemostasis” with bipolar cautery and injection of the flap suspended by tissue grasping forceps in an antigravitational manner.

“The Three Gorges” flap may be a valuable option or even a “life-boat” for a mega flap alongside the ALT-AMT and LD flap.

## Supplementary Material

Supplementary_Video_1_rjaf408
